# Prevalence and distribution of soil-transmitted helminth infections in Nigerian children: a systematic review and meta-analysis

**DOI:** 10.1186/s40249-018-0451-2

**Published:** 2018-07-09

**Authors:** Solomon Ngutor Karshima

**Affiliations:** Department of Veterinary Public Health and Preventive Medicine, University of Jos, PMB 2084, Jos, Nigeria

**Keywords:** Children, Distribution, Nigeria, Prevalence, Soil-transmitted helminth infections, Risk zones

## Abstract

**Background:**

Soil transmitted helminth (STH) infections still remain a notable health problem in resource-limited countries due to difficulties in the implementation of control measures. In Nigeria for instance, despite several community-based and provincial reports, national data on prevalence, burdens and risk zones (RZs) for STH infections are lacking.

**Methods:**

The present study employed the recommendations of the Preferred Reporting Items for Systematic Reviews and Meta-Analyses (PRISMA) to determine the prevalence, distribution and RZs for STH infections among Nigerian children through a meta-analysis of data published between 1980 and 2015. Pooled prevalence estimate (PPE) was determined by the random-effects model while heterogeneity was evaluated using the Cochran’s Q-test.

**Results:**

A total of 18 901 of the 34 518 Nigerian children aged 0–17 years examined across 19 Nigerian states during the period under review were infected with one or more species of STHs. The overall PPE for STH infections was 54.8% (95% *CI*: 54.2–55.3). PPEs for sub-groups ranged between 13.2% (95% *CI*: 11.5–15.1) and 80.9% (95% *CI*: 80.0–81.7). Highest PPEs for STH infections were observed among children within community settings (59.0%, 95% *CI*: 57.7–60.4) and school-aged children (54.9%, 95% *CI*: 54.3–55.5). *Ascaris lumbricoides* was the most prevalent species (44.6%, 95% *CI*: 44.0–45.2). Over 36% (15/41) of the studies were published from south-western Nigeria. South-western region was the only high risk zone (HRZ) for STH infections while the rest of the regions were low risk zones (LRZs).

**Conclusions:**

STH infections involving *Ascaris lumbricoides*, *Strongyloides stercoralis*, *Trichuris trichiura* and hookworms are highly prevalent across Nigeria. Strategic use of anthelmintics, health education and adequate sanitation, taking into account this epidemiologic information will help in the control of these infections in Nigeria.

**Electronic supplementary material:**

The online version of this article (10.1186/s40249-018-0451-2) contains supplementary material, which is available to authorized users.

## Multilingual abstract

Please see Additional file 1 for translations of the abstract into the five official working languages of the United Nations

## Background

Soil-transmitted helminths are among the leading causes of global health problems especially among the poorest and deprived communities where implementation of control measures is difficult [1, 2]. Globally, over one billion people are infected by at least one of the commonest species namely: *Ascaris lumbricoides* (the roundworm), *Trichuris trichiura* (the whipworm) *Strongyloides stercoralis* (threadworm) and the hookworms; *Ancylostoma duodenale* and *Necator americanus* [3].

Environmental survival of STH eggs and larvae including hatching and embryonation are determined by warm temperatures and adequate moisture [4]. Human infection is influenced by poverty, poor personal hygiene, inadequate sanitation and overcrowding [5, 6]. Infections may result in anaemia, retarded growth, and impaired cognitive development [7] and are classified among the major causes of absenteeism and disability adjusted life years lost [8].

Substantive evidence suggests that the most vulnerable group are children [4, 9] where infections are acquired through playing with contaminated soil and pica habits [10, 11]. Despite global decline in the prevalence of *A. lumbricoides*, *T. trichiura* and the hookworms (*A. duodenale* and *N. americanus*) in the Americas and Asia, the situation in sub-Saharan Africa remains stagnant [12].

According to the World Health Organization (WHO), administration of drugs like albendazole and mebendazole, health education and adequate sanitation are central to the control of STH infections. Community-based strategic drug administration which is vital to the control of STH infections requires epidemiological assessment and disease prevalence in communities as guides for choosing and instituting treatments [13].

Published literature on the prevalence of STH infections in Nigeria dates back to the 1970s [14]. However, there is no evidence of national control programmes despite advocacies for improved sanitation, health education and targeted chemotherapy in high risk communities to reduce the burden of these infections in Nigeria [15–18]. For instance, there have been some sporadic and uncoordinated deworming programmes, mostly sponsored by few politicians and philanthropists.

Recent STH control programmes focus on mass drug administration (MDA) in endemic regions to reduce parasite burdens and their effects [19, 20]. The successes and cost-effectiveness of these MDA programmes depend on the knowledge of STH prevalence which is used to classify communities into high or low RZs. In a resource-limited country like Nigeria, with a population of over 190 million people, cost-effectiveness in the control of STH infections is essential to ensure efficient allocation of resources and achievement of high impact. Hence, the aim of this meta-analysis which is the first of its kind in Nigeria was to provide useful epidemiological information including endemic species of STHs, their prevalence, distribution and RZs in Nigeria. This will serve as a guide for targeted control and ensure cost effective control of STH infections in Nigeria.

## Methods

### Country profile

**Fig. 1:**
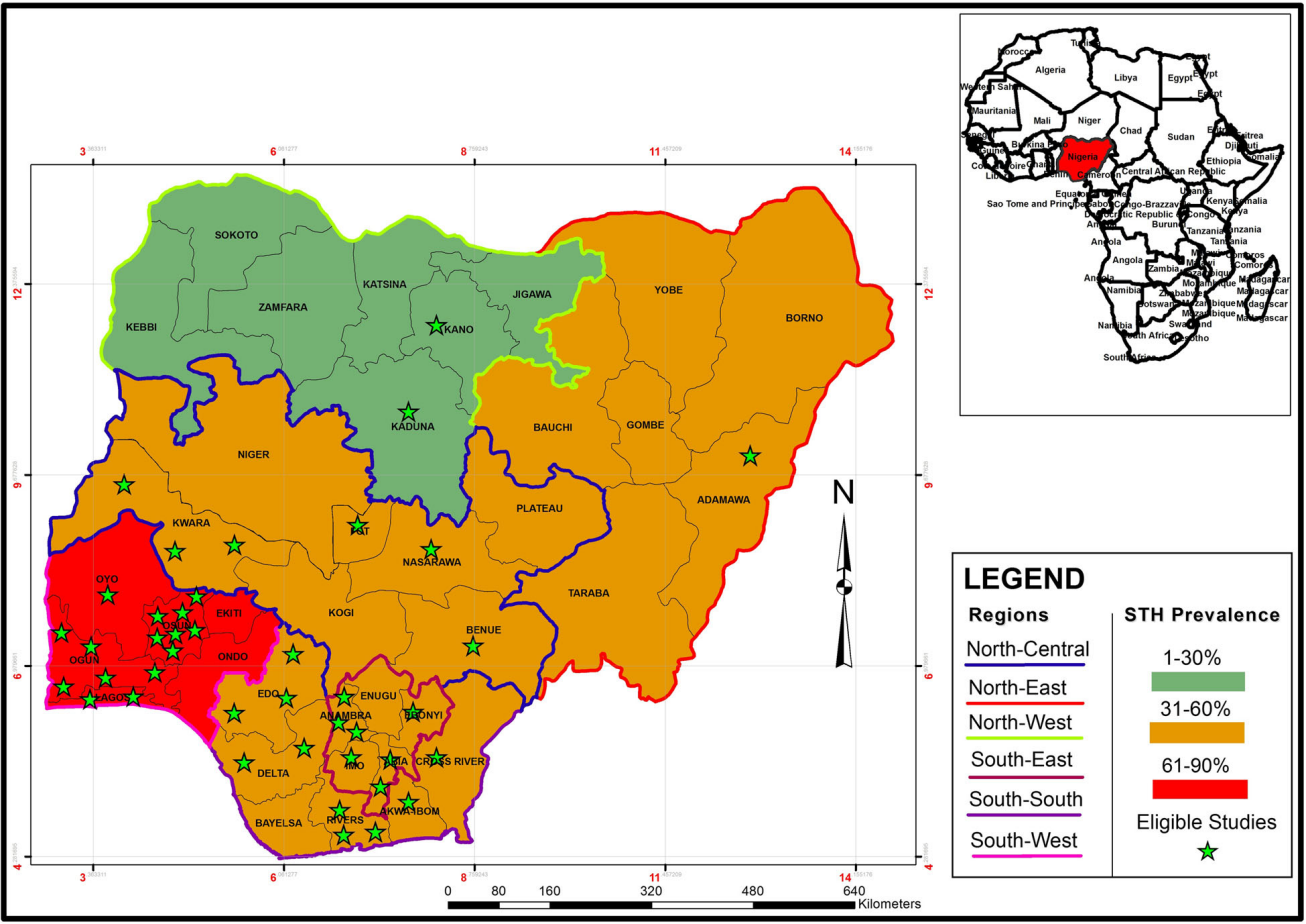
Study areas, regional prevalence and distribution of eligible studies

Studies included in this meta-analysis were carried out in Nigeria; a country with a population of over 190 million people which covers a surface area of 923 768 km^2^ in the sub-Saharan African region (Fig. 1). It has two distinct seasons; the rainy season which runs from March to November in the southern region and May to October in the northern region as well as the dry season which runs from December to February in the south and November to April in the North [21].

### Bibliography search strategy

The present study followed the PRISMA guidelines published by Moher et al. [22]. The study conducted a systematic review to identify studies that reported the prevalence of STH infections in Nigerian children. Data from suitable studies were then meta-analysed to determine pooled prevalence estimates (PPEs), distribution and RZs of STHs in Nigeria. Inclusion of information in the study was guided by the PRISMA checklist (Additional file 2). The outcome of interest was the presence of STH infections in Nigerian children.

Published studies were searched in four databases (PubMed, Google Scholars, Medline, AJOL) and lists of references of retrieved articles between September 2016 and March 2017. For clarity and ease of understanding and interpretation of contents, only studies published in English which is the official language in Nigeria, were included in the analysis. Because children are the most vulnerable group targeted by majority of STHs control programmes, selection process was restricted to studies carried out on children. To ensure that data included in the analysis were creditable and of good quality, only data published in reputable journals indexed at least in African Journals Online were included in the study. Since it was one of the objectives of the study to determine the prevalence and distribution of STH infections across Nigeria, study selection was restricted to studies with clearly stated sample sizes, number of positive samples and study locations.

Keywords employed for the literature search were: Prevalence/occurrence of soil-transmitted helminths/geo-helminths in Nigerian children. Common names of soil-transmitted helminths such as roundworm, whipworm, threadworm and hookworms were also used. Genera and species names for STHs of humans such as *Ascaris* ± *lumbricoides*, *Strongyloides* ± *stercoralis, Trichuris* ± *trichiura*, *Ancylostoma* ± *duodenale*, *Necator* ± *americanus* were also employed. Searches were narrowed down to regions like the north-central, north-east, north-west, south-east, south-south, south-west and the 36 states of the Nigerian federation.

### Criteria for inclusion and exclusion of studies

Studies were first screened through title review for relevance and removal of duplicates. This was followed by a detailed abstract and full text review to determine the presence of the outcome of interest and other inclusion requirements. Eligibility for inclusion of a study was based on the following conditions: (i) it was carried out in Nigeria, (ii) it was published in English, (iii) it was a cross sectional study, (iv) study location was clearly stated, (v) sample size and number of positive cases were clearly stated, (vi) it was published in a reputable journal indexed at least in African Journal Online, (vii) it reported STH infections in Nigerian children, (viii) parasites were identified at least to the genus level with the exception of hookworms. All the studies included in the analysis were assessed for quality independently using the Newcastle-Ottawa Scale according to the Cochrane Handbook for Systematic Reviews [23, 24].

### Data extraction

Data extracted from the eligible studies were: surname of first author, year of conduct and publication of study, sample size, number of positives cases, state and region of study, study design, species of STHs identified at least to the genus level.

### Data collation and analysis

Data were first entered through Microsoft Excel version 2007 (MS Corporation, Washington, USA) and further subjected to Graph-Pad Prism version 4.0 (Graph-Pad Software, San Diego, USA) and Comprehensive Meta-Analysis version 3.0 (Biostat, Englewood, USA) for statistical and meta-analysis respectively. Prevalence for individual studies was determined by multiplying the ratio of cases to sample size by 100. The 95% Confidence Interval (95% *CI*) was determined using the exact binomial interval (http://statpages.info/confint.html). Based on the assumption that true effect sizes might differ within eligible studies, the random-effects model was used to determine PPEs and their 95% *CI* [25]. Heterogeneity, which is the measure of variability between studies analysed was evaluated using the Cochran’s Q-test while percentage variation in prevalence estimate due to heterogeneity was quantified using the formula *I*^*2*^ = 100 × (Q-df)/Q, where Q is Cochran’s heterogeneity statistic and *df* is the degree of freedom which is the difference between the number of studies and one. *I*-square values of 0, 25, 50 and 75% were considered as no, low, moderate and high heterogeneities respectively [26]. RZs for STH infections were categorized based on prevalence of infections as recommended by the WHO. Regions with PPEs ≤20% to < 50% were classified as LRZs while regions with PPEs ≥50 were classified as HRZs for STH infections [27].

## Results

### Literature search and eligible studies

**Fig. 2:**
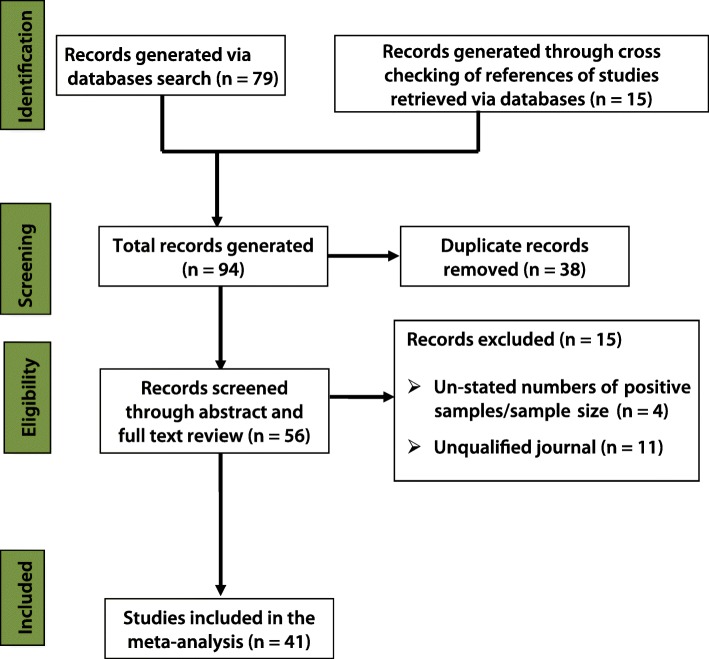
Flow diagram for the selection process of eligible studies

The selection process for eligible studies is presented in Fig. 2. Of the 94 studies retrieved, 79 and 15 were generated through the search of databases and lists of references of articles respectively. Thirty eight duplicate studies were removed after the screening of titles. Fifty six studies were further subjected to abstract and full text review where 15 studies were excluded for the following reasons: unstated numbers of positive samples and sample sizes (*n* = 4) and quality of publishing journals (*n* = 11).

### Characteristics of the eligible studies

Table 1 presents the characteristics of the studies meta-analysed. Forty one studies were eligible and thus were included in the analysis. Studies were conducted between 1980 and 2014 and published between 1981 and 2015. Four, seven and 30 of the studies were carried out between 1980 and 1991, 1992 and 2003 as well as 2004 and 2014 respectively. One, two, six, seven, 11 and 14 of the studies were reported from north-east, north-west, north-central, south-east, south-south and south-west Nigeria respectively. Two, nine and 30 studies were carried out within hospital, community and school settings respectively. Five, six, seven and 23 of the studies had sample sizes of greater than 1500, 501–1000, 1001–1500 and 100–500 respectively. Thirty three studies were reported among school-aged children while 8 of the studies were reported among pre-school-aged children. Prevalence of STH infections among eligible studies ranged between 6.0 and 96.1%.

**Table 1:** List and characteristics of the 41 eligible studies

Study ID	Year of study	State	Region	Study setting	Sample size	Cases	Prevalence (%)	95% *CI*
[57]	2011	Rivers	SSR	School-based	3826	1050	27.4	26.0–28.9
[58]	2012/2013	Kwara	NCR	School-based	1017	229	22.5	20.0–25.2
[59]	1989	Kwara	NCR	Community-based	907	797	87.9	85.6–89.9
[60]	2002	Ogun	SWR	School-based	1253	1129	90.1	88.3–91.7
[61]	2006	Lagos	SWR	School-based	1177	579	49.2	46.3–52.1
[62]	2005/2006	Ogun	SWR	School-based	1059	872	82.3	79.9–84.6
[63]	2007	Cross River	SSR	Community-based	350	174	49.7	44.4–55.1
[64]	1985	Rivers	SSR	Community-based	1062	1020	96.1	94.7–97.1
[65]	2011	Anambra	SER	School-based	200	80	40.0	33.2–47.2
[66]	2014	Kwara	NCR	School-based	304	54	17.8	13.6–22.5
[67]	1980	Lagos	SWR	School-based	5595	4241	75.8	74.7–76.9
[68]	2008	Nasarawa	NCR	School-based	480	314	65.4	61.0–69.7
[69]	2004/2005	Ogun	SWR	School-based	232	112	48.3	41.7–54.9
[70]	1993	Anambra	SER	School-based	1536	775	50.5	47.9–53.0
[71]	1987	Oyo	SWR	School-based	766	678	88.5	86.0–90.7
[72]	2012	Kano	NWR	School-based	570	95	16.7	13.7–20.0
[73]	2005	Osun	SWR	Community-based	369	48	13.0	9.8–16.9
[74]	2006/2007	Osun	SWR	Community-based	1228	684	55.7	52.9–58.5
[75]	2005/2006	Delta	SSR	School-based	1200	960	80.0	77.6–82.2
[76]	2002	Kaduna	NWR	School-based	800	86	10.8	8.7–13.1
[77]	2011	Abuja	NCR	School-based	220	90	40.9	34.4–47.7
[78]	2011	Edo	SSR	Community-based	140	29	20.7	14.3–28.4
[79]	2008/2009	Edo	SSR	Hospital-based	310	170	54.8	49.1–60.5
[80]	2012	Delta	SSR	School-based	200	117	58.5	51.3–65.4
[81]	2007	Anambra	SER	School-based	514	275	53.5	49.1–57.9
[82]	2012	Imo	SER	School-based	284	88	31.0	25.7–36.7
[83]	2000	Osun	SWR	School-based	749	245	32.7	29.4–36.2
[84]	2009	Akwa Ibom	SSR	School-based	405	286	70.6	65.9–75.0
[85]	2005	Adamawa	NER	School-based	250	114	45.6	39.3–52.0
[86]	2013	Abia	SER	School-based	200	41	20.5	15.1–26.8
[87]	2003	Ogun	SWR	School-based	2837	1376	48.5	46.7–50.4
[88]	2003–2005	Ogun	SWR	School-based	1519	435	28.6	26.4–31.0
[89]	2009	Osun	SWR	Community-based	352	121	34.4	29.4–39.6
[90]	2005	Osun	SWR	Community-based	300	18	6.0	3.6–9.3
[91]	2012	Ebonyi	SER	School-based	300	244	81.3	76.5–85.6
[92]	2004/2005	Osun	SWR	School-based	489	468	95.7	93.5–97.3
[93]	2011	Osun	SWR	School-based	419	401	95.7	93.3–97.4
[94]	2002	Abia	SER	School-based	300	277	92.3	88.7–95.1
[95]	2014	Benue	NCR	Community-based	228	23	10.1	6.5–14.8
[96]	2001/2002	Edo	SSR	Hospital-based	207	44	21.3	15.9–27.5
[97]	2013	Rivers	SSR	School-based	364	62	17.0	13.3–21.3

*CI* Confidence interval, *NCR* North-central region, *NER* North-east region, *NWR* North-west region, *SER* South-east region, *SSR* South-south region, *SWR* South-west region

### Pooled prevalence estimates and heterogeneity analysis

**Fig. 3:**
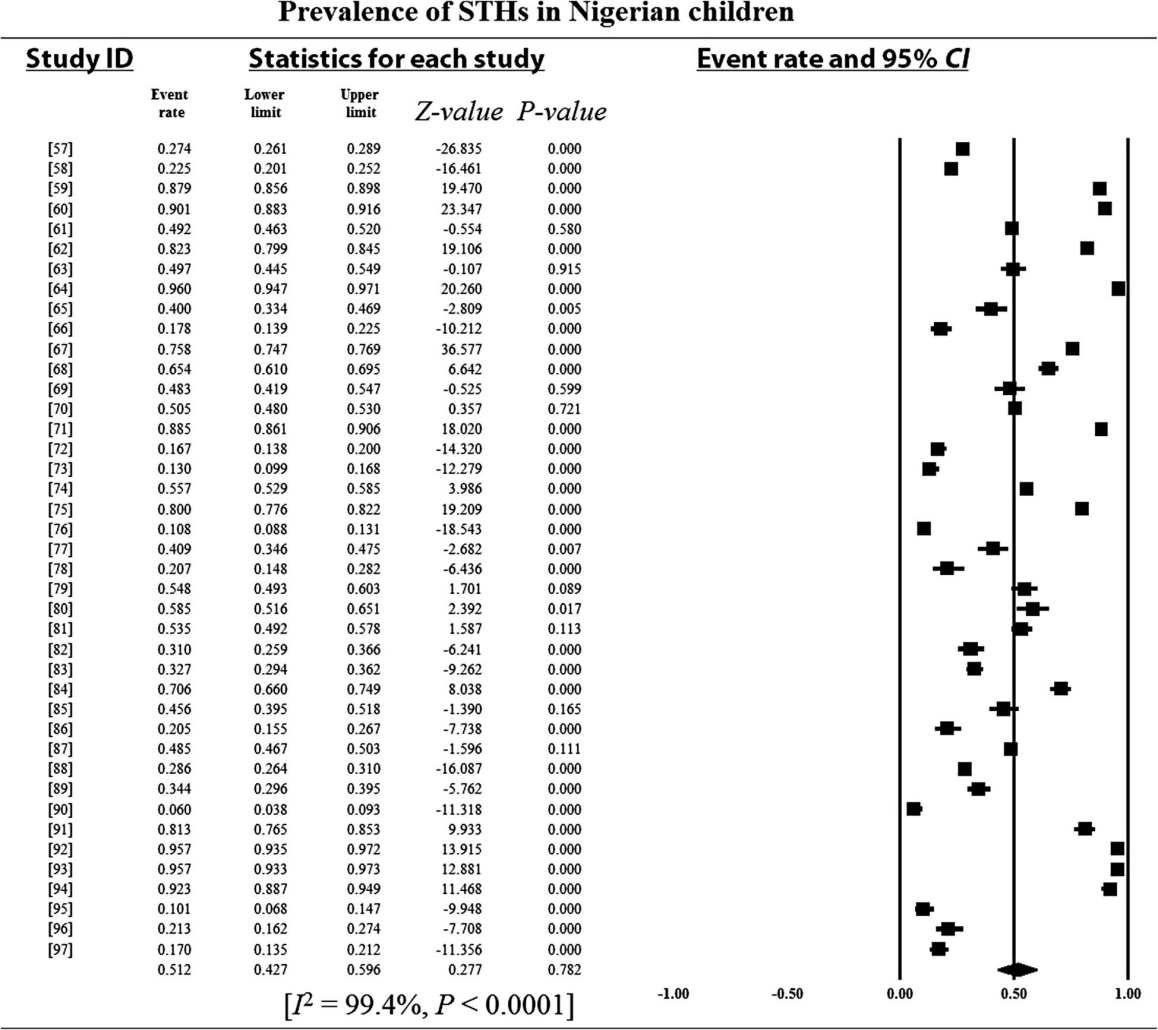
Forest plot for the prevalence of STHs in Nigerian children

**Fig. 4:**
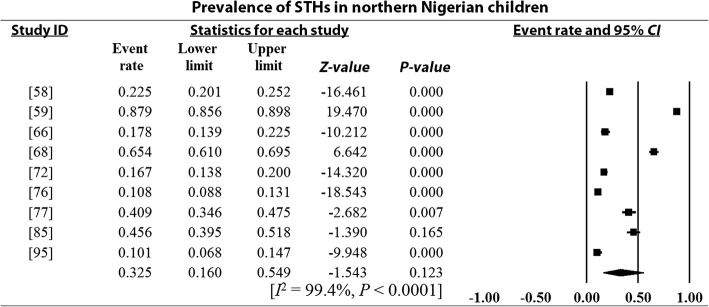
Forest plot for the prevalence of STHs in children from northern Nigeria

**Fig. 5:**
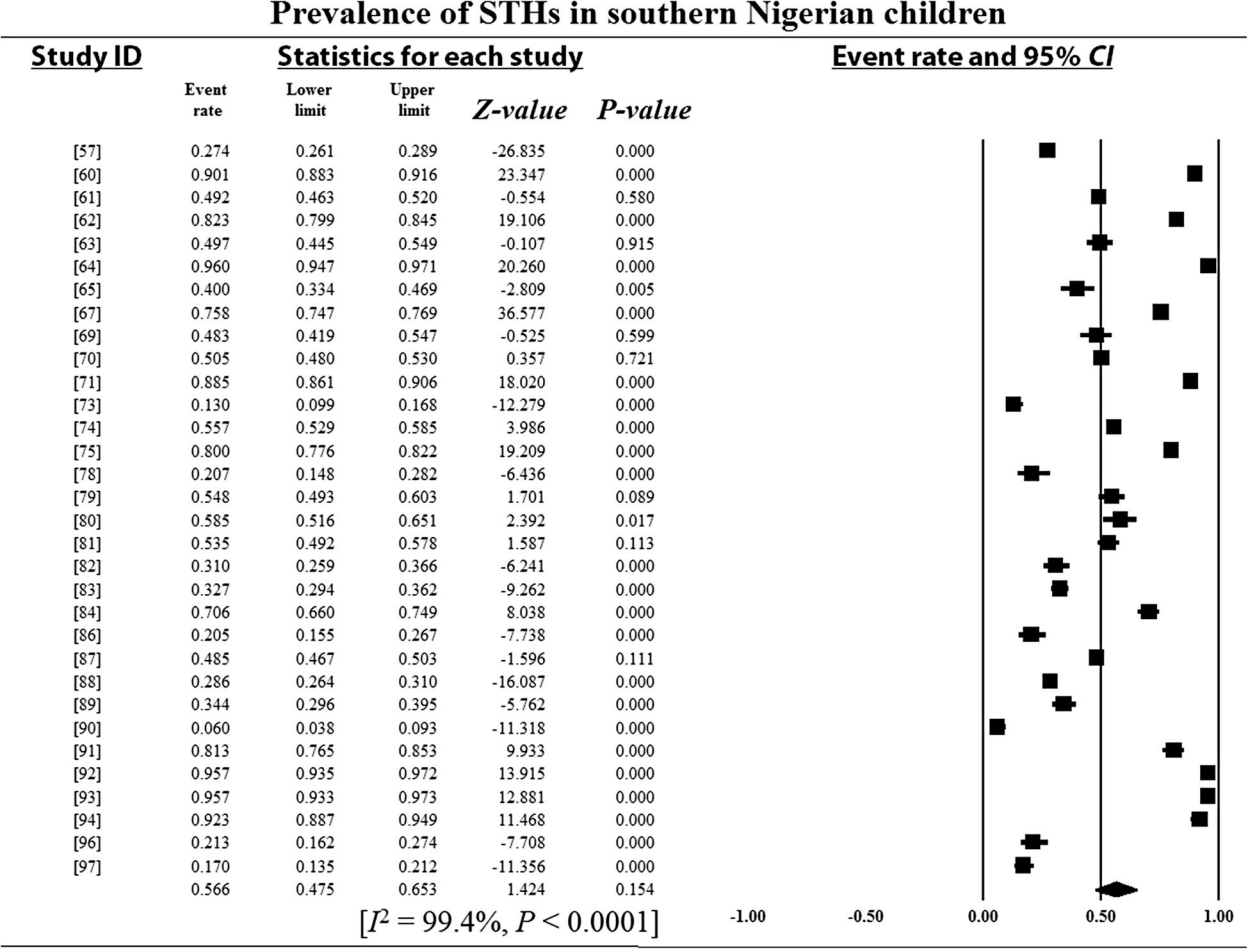
Forest plot for the prevalence of STHs in children from southern Nigeria

**Fig. 6:**
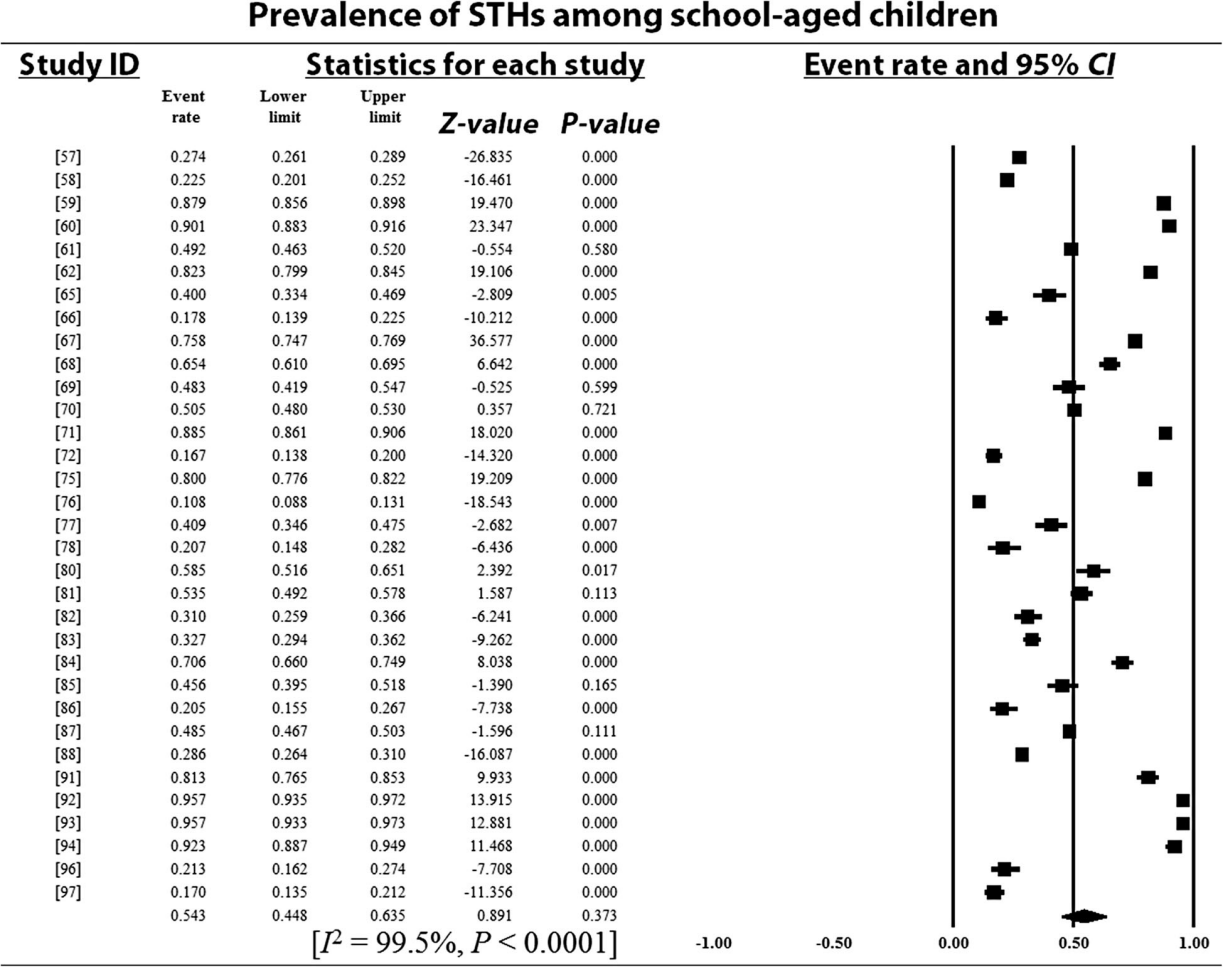
Forest plot for the prevalence of STHs among school-aged children

**Fig. 7:**
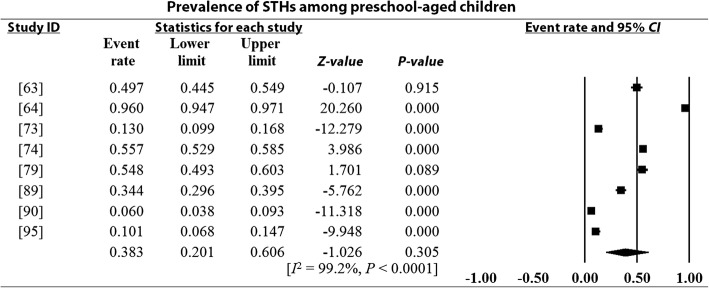
Forest plot for the prevalence of STHs among preschool-aged children

Overall and sub-group PPEs for STH infections are presented in Table 2. A total of 18 901 of the 34 518 Nigerian children examined during the period under review were infected with one or more species of STHs yielding an overall PPE of 54.8% (95% *CI*: 54.2–55.3). PPEs for sub-groups (regions, study period, sample size, study settings and school/preschool-aged children) ranged between 13.2% (95% *CI*: 11.5–15.1) and 80.9% (95% *CI*: 80.0–81.7). A high degree of heterogeneity 99.4% (95% *CI*: 54.2–5.3, *P* < 0.0001) was observed within studies and sub-groups (Figs. 3, 4, 5, 6 and 7 and Additional files 3, 4 and 5).

**Table 2:** Pooled prevalence estimates for STH infections in Nigerian children stratified according to sub-groups

Variables	No. of studies	Pooled prevalence estimates			95% *CI*	Heterogeneity	
		Sample size	Positives	Prevalence (%)		*I*^*2*^ (%)	Q-*P*
Region							
North-central	6	3156	1507	47.8	46.0–49.5	99.4	0.000
North-east	1	250	114	45.6	39.3–52.0	0.0	0.000
North-west	2	1370	181	13.2	11.5–15.1	90.0	0.002
South-east	7	4653	2174	46.7	45.3–48.2	99.4	0.000
South-south	10	8264	3953	47.8	46.8–48.9	98.8	0.000
South-west	15	16 825	10 972	65.2	64.5–65.9	99.5	0.000
Study period							
1980–1991	4	8330	6736	80.9	80.0–81.7	98.9	0.000
1992–2003	7	7682	3932	51.2	50.1–52.3	99.5	0.000
2004–2014	30	18 506	8233	44.5	43.8–45.2	99.1	0.000
Sample size							
100–500	23	6903	3375	48.9	47.7–50.1	98.6	0.000
501–1000	6	4306	2176	50.5	49.3–52.0	99.6	0.000
1001–1500	7	7996	5473	68.5	67.4–69.5	99.6	0.000
> 1500	5	15 313	7877	51.4	50.7–52.2	99.8	0.000
Study setting							
Community-based	9	4936	2914	59.0	57.7–60.4	99.4	0.000
Hospital-based	2	517	214	41.4	37.1–45.8	99.1	0.000
School-based	30	29 065	15 773	54.3	53.7–54.8	99.5	0.000
SPSAG							
SAC	33	30 319	16 643	54.9	54.3–55.5	99.5	0.000
PSAC	8	4199	2258	53.8	52.3–55.3	99.2	0.000
Overall	41	34 518	18 901	54.8	54.2–55.3	99.4	0.000

*CI* Confidence interval, *I*^*2*^ Inverse variance index, Q-*P* Cochran’s *P*-value, *SPSAG* School/Preschool-age groups, *SAC* School-aged children, *PSAC* Preschool-aged children

*A. lumbricoides* had the highest PPE of 44.6% (95% *CI*: 44.0–45.2) while, *T. trichiura*, hookworms and *S. stercoralis* recorded PPEs of 31.9% (95% *CI*: 31.3–32.5), 23.0% (95% *CI*: 22.5–23.5) and 3.4% (95% *CI*: 3.1–3.8) respectively (Table 3). *A. lumbricoides* (53.9%) and *T. trichiura* (43.8%) were the most prevalent species in the south-west while, hookworms (34.4%) and *S. stercoralis* (10.9%) recorded the highest prevalence in north-east and north-central regions respectively (Table 4). The highest prevalence of *A. lumbricoides* (46.6%), hookworms (27.3%) and *T. trichiura* (36.5%) were observed among school-children while, *S. stercoralis* recorded the highest prevalence among children sampled within the community (Table 4).

**Table 3:** Species-specific pooled prevalence estimates for STH infections

Parasites	Number of studies	Pooled prevalence estimates			95% *CI*	Heterogeneity	
		Sample size	Positives	Prevalence (%)		*I*^*2*^ (%)	Q-*P*
*Ascaris lumbricoides*	38	29 177	13 006	44.6	44.0–45.2	99.4	0.000
Hookworms	34	25 634	5898	23.0	22.5–23.5	99.5	0.000
*Strongyloides stercoralis*	15	10 581	364	3.4	3.1–3.8	99.5	0.000
*Trichuris trichiura*	31	23 089	7373	31.9	31.3–32.5	99.4	0.000

*STH* Soil-transmitted helminths, *CI* Confidence interval, *I*^*2*^ Inverse variance index, Q-*P* Cochran’s *P*-value

**Table 4:** Pooled prevalence estimates for STH infections in relation to regions and study settings

	*Ascaris lumbricoides*		Hookworms		*Strongyloides stercoralis*		*Trichuris trichiura*	
Variables	SSZ	Cases (%)	SSZ	Cases (%)	SSZ	Cases (%)	SSZ	Cases (%)
Regions								
North-central	1935	596 (30.8)	2936	476 (16.2)	1387	151 (10.9)	955	308 (32.3)
North-east	250	57 (22.8)	250	86 (34.4)	250	23 (9.2)	250	26 (10.4)
North-west	1370	119 (8.7)	570	30 (5.3)	800	12 (1.5)	570	20 (3.5)
Total	3555	772 (21.7)	3756	592 (15.8)	2437	186 (7.6)	1775	354 (19.9)
South-east	3334	918 (27.5)	3334	898 (26.9)	2250	78 (3.5)	2850	335 (11.8)
South-south	5297	2111 (39.9)	5157	848 (16.4)	2892	106 (3.7)	5297	1144 (21.6)
South-west	17 091	9205 (53.9)	11 289	3549 (31.5)	3002	34 (1.1)	12 638	5540 (43.8)
Total	25 722	12 234 (47.6)	19 780	5295 (26.8)	8144	218 (2.7)	20 785	7019 (33.8)
Study setting								
Community	4936	1828 (37.0)	4427	200 (4.5)	1969	99 (5.0)	4708	761 (16.2)
Hospital	517	133 (25.7)	517	42 (8.1)	207	1 (0.5)	517	38 (7.4)
School	23 724	11 045 (46.6)	20 690	5656 (27.3)	8405	321 (3.8)	17 867	6517 (36.5)
Total	29 177	13 006 (44.6)	25 634	5898 (23.0)	10 581	421 (4.0)	23 092	7316 (31.7)

*SSZ* Sample size

### Regional distribution of eligible studies and RZs for STH infections

**Fig. 8:**
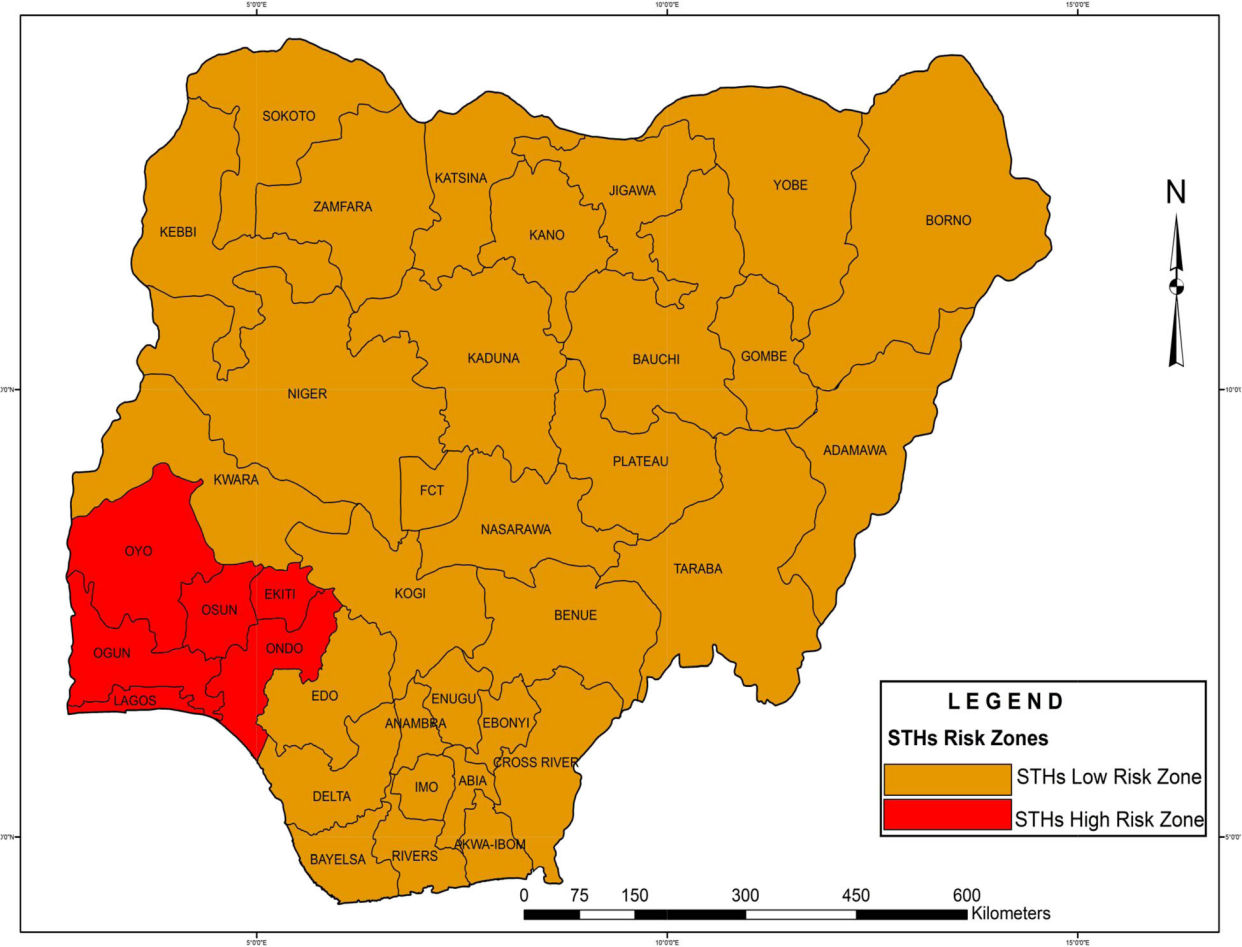
Risk zones for soil-transmitted helminth infections

The distribution of eligible studies is presented in Fig. 1. The highest numbers of studies were reported in the south-west region: 15 (46.3%) and Osun State: seven (17.1%). These were followed by the south-south region with 10 (24.4%) and Ogun State with five (12.2%). The south-west region recorded STH prevalence of 65.2% and is classified as HRZ while the rest of the regions recorded prevalence estimates ranging between 13.2 and 47.8% and are classified as LRZs (Fig. 8).

## Discussion

The present study was designed to complement global efforts towards the control of neglected tropical diseases by providing useful epidemiological data that will aid their control. The study provides information on endemic species of STHs, their national and regional prevalence, their distribution in relation to regions, species, periods and settings as well as RZs. This data is essential because strategic anthelmintic control of STHs depends on community-based prevalence [28]. The findings will also (i) help in assessing successes of sporadic STHs control programmes in Nigeria which usually target children and (ii) provide information that will serve as a guide for targeted and cost-effective control which is a subject of debate globally [29–32].

The overall pooled prevalence estimate (54.8%) observed in the present study is higher than the 24.1% [33] and 25.4% [34] reported in Cameroon and Rwanda respectively. The finding is however within the range of 52.4–65.8% reported from other sub-Saharan African countries [35, 36]. These variations may be attributable to differences in environmental factors such as temperature, humidity, rainfall [37] and soil moisture [38]. Other factors may be differences in levels of hygiene and sanitation, environmental contamination [39] as well as the specificity and sensitivity of the diagnostic methods employed by the individual studies.

The forested nature, the high level of rainfall, low humidity and temperature in south-western Nigeria [21] may explain the higher prevalence (65.2%) of STH infections in the region. This suggests that this region may be the most endemic for STHs in Nigeria. Since cost-effective control requires knowledge of community prevalence for correct choice of anthelmintic strategy, this information may be useful for stakeholders in STHs control. The lower prevalence reported in the north-western region may be attributable to the extremely high temperatures in these regions [21] which may not support environmental survival of eggs and larvae of these parasites [4].

The study revealed a 36.4% decline in the prevalence of STH infections within a period of twenty four years. This may not be unconnected with the global campaigns targeting eradication of neglected tropical diseases by year 2020 [40] resulting in increased efforts towards the control of STH infections in recent times. The higher number of studies reported during the most recent decade may be attributable to increased awareness of the public health threats posed by these parasites.

Studies carried out in communities other than schools and hospitals recorded the highest PPE probably due to the sporadic STHs control programmes in Nigeria which usually targets school and hospitalized children. Though there are scanty reports of STH infections among adults in Nigeria, the PPE reported in the present study is higher than the range of 9.4–28.6% [41–43] documented among adults in Nigeria. Reports of STH infections in adults in Nigeria and other sub-Saharan African countries [44–46] suggest that the burden of these parasites is not restricted to children. These findings suggest the need for Nigeria to adopt the current WHO recommended strategy for the control of STH infections at community levels. This strategy involves prevalence-based targeted distribution of albendazole and mebendazole in both school and preschool-aged children as well as women of child bearing age [40].

The species of STHs reported in Nigerian children during the period under review are similar to those reported in other sub-Saharan African countries like Cameroon [47], Ethiopia [36], Kenya [48] and Uganda [49]. This finding shows that these parasites are still endemic in the region suggesting that extra efforts are required to achieve the WHO’s goal of eradication in sub-Saharan Africa by 2020.

*A. lumbricoides* was the most prevalent species of STHs reported during the period under review while, hookworms had the lowest prevalence in agreement with global data [2, 3, 12]. The high prevalence of *A. lumbricoides* observed by the present study may be attributable to high environmental contamination resulting from the large number of infected people [4], the durability of *Ascaris* eggs under varying environmental conditions [50], the high fecundity [51] as well as the sticky nature of the shell of *Ascaris* egg [52] which aids its attachment on human hands, fruits and vegetables.

South-western Nigeria recorded the highest PPE for *A. lumbricoides* (53.9%) and *T. trichiura* (43.8%) probably due to the forested nature, high rainfall, low humidity and temperature [21] in this region. The highest prevalence of hookworms in the north-eastern region may not be unconnected with the practice of inhabitants of the region walking barefoot [5, 6]. Other possible factors responsible for the high prevalence of these species in the region may include poverty, inadequate sanitation, overcrowding and the consumption of unwashed fruits and vegetables [53].

The rainy season influences soil moisture. This in turn determines the survival of STH eggs and larvae in the environment. Public education on the high risk of acquiring infection during this season especially through the consumption of contaminated vegetables will be a good measure towards the control of these infections. Since this season is associated with high intensity of STH infections, MDA campaigns should be programmed to target the rainy season for effectiveness.

Though the sporadic control efforts in Nigeria usually target school-aged children, the present study revealed a similar prevalence between school and preschool-aged children indicating possible failures in these control programmes. It is envisaged that the nation will take advantage of the information provided on regional and national prevalence, distribution and RZs for STH infections to re-strategize on their control in Nigeria.

A recent study in Nigeria by Oluwole et al. [54] which utilized data produced by a large scale national survey conducted by the Ministry of Health among children aged 5–14 years across the country observed prevalence of > 50% for *A. lumbricoides* and hookworms and a range of 20.01–50.00% for *T. trichiura* in several locations within the south-western region. The same study showed that most locations in the north-central, north-eastern, north-western, south-eastern and south-south regions reported the range of < 1.00–20.00% while a few locations had prevalence range of 20.01–50.00%. Their report supports the classification of the north-central, north-east, north-west, south-east and south-south as LRZs and south-west as HRZ by the present study. These two concurring reports suggest that the majority of Nigerian regions are LRZs for STH infections.

The idea behind the present study is to complement global efforts towards elimination of STH infections by 2020 as targeted by the WHO and the London declaration [55, 56]. This finding will therefore be a guide for instituting national MDA programmes which classify communities into low risk (prevalence: 20% to < 50%) and high risk (prevalence ≥50%) and their respective treatment regimens. The present finding shows that the south-western region which has a PPE of 65.2% is a high risk community while the rest of the regions recorded PPEs ranging between 13.2 and 47.8% and are considered low risk communities. Based on the WHO guidelines for MDA, biannual albendazole or mebendazole treatment is recommended for people living in the south-west Nigeria (prevalence: 65.2%) while single annual treatment is recommended for people living in the north-central, north-east, south-east and south-south Nigeria (prevalence: 45.6–47.8%). For people living in the north-western region where prevalence was less than 20% (13.2%), improved sanitation, health education and a case-by-case handling of affected individuals [27] is recommended. Effective implementation of this programme which is highly cost effective will drastically reduce the burden of STH infections in Nigeria.

Despite the valuable data provided by this study, it is not devoid of limitations. Studies were reported from only 19 of the 36 states including the Federal Capital Territory. Studies were unevenly distributed across regions, study period and study settings. Other studies which would have added to our understanding of STHs situation in Nigeria were excluded for incomplete information. The study revealed high heterogeneity among studies which may be due to variations in study designs, methodologies, sample populations and methods of diagnosis employed by the various studies.

## Conclusions

STH infections are highly prevalent and well distributed across Nigeria and within community, hospital and school settings. *A. lumbricoides* was the most prevalent of the STH species. The south-west is a HRZ for STH infections while the rest of the regions are LRZs. The adoption of the current WHO recommended strategy for the control of STH infections at community levels which involves prevalence-based targeted distribution of albendazole and mebendazole among school and preschool-aged children, women of child-bearing age and adults in RZs in Nigeria will reduce the menace pose by these parasites.

## Additional files

Additional file 1: Multilingual abstracts in the five official working languages of the United Nations. (PDF 234 kb)

Additional file 2: Preferred Reporting Items for Systematic Reviews and Meta-Analyses (PRISMA) checklist. (DOC 62 kb)

Additional file 3: Forest plot for the prevalence of STHs in children sampled within communities. (DOCX 77 kb)

Additional file 4: Forest plot for the prevalence of STHs in children sampled within schools. (DOCX 95 kb)

Additional file 5: Forest plot for the prevalence of STHs in children sampled within hospitals. (DOCX 50 kb)

## Abbreviations

AJOL: African Journals OnLine
*CI*: Confidence interval
df: Degree of freedom
HRZ(s): High risk zone(s)
*I*^2^: Inverse variance index
ID: Identification
LRZ(s): Low risk zone(s)
MDA: Mass drug administration
NCR: North-central region
NER: North-east region
NWR: North-west region
PPE(s): Pooled prevalence estimate(s)
PRISMA: Preferred Reporting Items for Systematic Reviews and Meta-Analyses
PSAC: Preschool-aged children
Q: Cochran’s heterogeneity statistics
Q-p: Cochran’s *P*-value
RZ(s): Risk zone(s)
SAC: School-aged children
SER: South-east region
SPSAG: School/Preschool-age groups
SSR: South-south region
SSZ: Sample size
STH: Soil-transmitted helminths
SWR: South-west region
WHO: World Health Organization
