# Supplementary material for: Prevalence and distribution of soil-transmitted helminth infections in Nigerian children: a systematic review and meta-analysis
Source: Infect Dis Poverty. 2018 Jul 9;7:69. doi: 10.1186/s40249-018-0451-2 (PMC6036687; doi:10.1186/s40249-018-0451-2)
Supplement: Supplementary file 1 — Multilingual abstracts in the five official working languages of the United Nations. (PDF 234 kb) [file 40249_2018_451_MOESM1_ESM.pdf]

Translation of the abstract into the five official working languages of the United Nations

انتشار وتوزيع عدوى الديدان الطفيلية المنقولة بالتربة في الأطفال النيجيريين: مراجعة منهجية وتحليل تلوي

سولومون نغوتور كارشيما

الملخص

الخلفية: لا تزال إصابات الديدان الطفيلية المنقولة عن طريق التربة (STH) تمثل مشكلة صحية ملحوظة في البلدان محدودة الموارد بسبب الصعوبات في تنفيذ تدابير المكافحة. فعلى سبيل المثال في نيجيريا، وعلى الرغم من العديد من التقارير المجتمعية والإقليمية، إلا أن هنالك نقصا في البيانات القومية عن انتشار وأعباء ومناطق الخطر (RZs) لإصابات الديدان الطفيلية المنقولة عن طريق التربة.

الطرق : استخدمت الدراسة الحالية توصيات عناصر الإبلاغ المفضلة للمراجعات المنهجية والتحليل التلوي (PRISMA) لتحديد مدى انتشار، وتوزيع ومناطق الخطر RZs لعدوى الديدان الطفيلية المنقولة عن طريق التربة STH بين الأطفال النيجيريين من خلال التحليل التلوي للبيانات المنشورة بين 1980 و 2015. تم تحديد تقدير الانتشار المجمّع (PPE) عن طريق نموذج التأثيرات العشوائية بينما تم تقييم عدم التجانس باستخدام اختبار كوكران Cochran’s Q-test

النتائج : تم إحصاء ما مجموعه 901 18 من الأطفال النيجيريين البالغ عددهم 34518 طفلاً تتراوح أعمارهم بين 0-17 سنة في 19 ولاية نيجيرية خلال الفترة قيد الاستعراض مصابين بنوع واحد أو أكثر من أنواع الديدان الطفيلية المنقولة عن طريق التربة STHs. معدل الانتشار المجمَّع لعدوى الديدان الطفيلية المنقولة بالتربة كان 54.8 (95٪ نطاق الثقة: 54.2 - 55.3). تراوحت معدّلات الانتشار المجمَّع للمجموعات الفرعية بين 13.2% (95% نطاق الثقة: 11.5, 15.1) و 80.9% (95% نطاق الثقة : 80.0، 81.7). ولوحظت أعلى معدلات الانتشار المجمّع لعدوى الديدان الطفيلية المنقولة بالتربة STH بين الأطفال في محيط المجتمع (59.0%, 95% نطاق الثقة : 57.7, 60.4) والأطفال في سن المدرسة (54.9%, 95% نطاق الثقة: 54.3, 55.5). ثعبان البطن هو أكثر الأنواع انتشارًا (44.6٪ ، 95٪ نطاق الثقة : 44.0, 45.2). تم نشر أكثر من 36 ٪ (41/15) من الدراسات من جنوب غرب نيجيريا. كانت المنطقة الجنوبية الغربية هي المنطقة الوحيدة عالية المخاطر (HRZ) بالنسبة للإصابة بعدوى الديدان الطفيلية المنقولة بالتربة STH بينما كانت بقية المناطق مناطق منخفضة المخاطر (LRZs).

الخلاصة : تنتشر بصورة شديدة العدوى التي تنتقل عن طريق الديدان الطفيلية المنقولة بالتربة STH والتي تشمل ثعبان البطن , والاسطوانية البرازية , والدودة السوطية والأنكلستوما في نيجيريا. الاستخدام الاستراتيجي لطاردات الديدان، والتثقيف الصحي والصرف الصحي المناسب، مع الأخذ في الاعتبار هذه المعلومات الوبائية سيساعد في السيطرة على هذه العدوى في نيجيريا.

Translated from English version into Arabic by Free bird and Amjad Basheer, through

TRANSLATORS WITHOUT BORDERS

尼日利亚未成年土源性线虫感染者的流行情况和分布:系统综述和 meta 分析

Solomon Ngutor Karshima

摘要

引言：在医疗资源匮乏的国家中，由于难以开展控制措施，土源性线虫（STH）感染仍然是一个需要关注的健康问题。例如，在尼日利亚，尽管有一些基于社区和省级的报告，但仍然缺乏关于 STH 感染的疾病情况、负担和危险区域(RZs)的国家级数据。

方法：本研究根据系统综述和 meta 分析(PRISMA)首选报告项目的建议，通过在 1980 年至

2015 年间发表的数据进行 meta 分析，以确定尼日利亚未成年人感染的流行情况、分布和 RZs。采用随机效应模型决定混合流行估计值(PPE)，并使用 Cochran’s Q-test 检验来评估其异质性。
结果：在本研究期间，对 19 个尼日利亚州的 34,518 未成年人进行了调查，其中有 18,901 人感染了一种或多种类型的 STHs。感染的总 PPE 为 54.8% (95% *CI*: 54.2–55.3)。亚组的 PPEs 介于 13.2% (95% *CI*: 11.5–15.1)和 80.9% (95% *CI*: 80.0–81.7)之间。在社区中生活的儿童(59.0%，95% *CI*: 57.7–60.4)和学龄儿童(54.9%，95% *CI*: 54.3–55.5) 的感染率最高。最常见的感染虫种是蛔虫(44.6%，95% *CI*: 44.0–45.2)。超过 36%(15/41)的研究来源于尼日利亚西南部。西南地区是 STHs 传播的唯一高危险区(HRZ)，而其它地区为低危险区(LRZs)。
结论：在尼日利亚，STH 感染，包括蛔虫、大叶虫、三叶虫和钩虫感染的流行率均较高。基于这一流行病学信息，进而战略性地使用驱虫剂、开展健康教育和足够的卫生清洁将有助于控制这些感染。

Translated from English version into Chinese by Xin-Yu Feng, edited by Jin Chen, through
CHINESE CENTER FOR DISEASE CONTROL AND PREVENTION
NATIONAL INSTITUTE OF PARASITIC DISEASES

Prévalence et distribution des géohelminthiases parmi les enfants nigérians : revue systématique et méta-analyse

Solomon Ngutor Karshima

Résumé
Contexte : Les géohelminthiases restent un problème considérable de santé publique dans les pays dont les ressources sont limitées et qui ont donc du mal à mettre en place des mesures de lutte contre ces parasitoses. Au Nigeria, par exemple, bien que plusieurs bilans aient été dressés au niveau de communautés ou de provinces, les données nationales sur la prévalence, le fardeau des géohelminthiases et les zones à risque sont insuffisantes.
Méthodes : La présente étude s’est appuyée sur les recommandations PRISMA (Preferred Reporting Items for Systematic Review and Meta-Analyses) pour déterminer la prévalence des HTS parmi les enfants nigérians, leur distribution et les zones à risque. À partir d’une méta-analyse des données publiées entre 1980 et 2015, nous avons procédé à une estimation de la prévalence groupée (PGE) à l’aide du modèle à effets aléatoires et évalué l’hétérogénéité à l’aide du test Q de Cochran.
Résultats : Au total, 18 901 des 34 518 enfants âgés de 0 à 17 ans examinés dans 19 états du Nigeria pendant la période de la revue étaient infestés par une ou plusieurs espèces d’HTS. La PGE globale des géohelminthiases était de 54,8% (IC à 95 % : de 54,2 à 55,3). Les PGE des sous-groupes allaient de 13,2 % (IC à 95 % : de 11,5 à 15,1) à 80,9 % (IC à 95 % : de 80,0 à 81,7). Les PGE les plus élevées pour les géohelminthiases ont été relevées parmi les enfants vivant en milieu communautaire (59,0 %, IC à 95 % de 57,7 à 60,4) et les enfants d’âge scolaire (54,9 %, IC à 95 % de 54,3 à 55,5). *Ascaris lumbricoides* était l’espèce la plus prévalente (44,6 %, IC à 95 % de 44,0 à 45,2). Plus de 36 % des études publiées (15 sur 41) ont été réalisées dans le sud-ouest du Nigeria, seule zone à haut risque de géohelminthiase tandis que les autres régions étaient des zones à bas risque.
Conclusion : Les géohelminthiases, notamment causées par *Ascaris lumbricoides*, *Strongyloides*

stercoralis, *Trichuris trichiura* et les ankylostomes, sont très prévalentes dans toute le Nigeria. L'utilisation stratégique des antihelminthiques, l'éducation à la santé et un assainissement adéquat aideront à lutter contre ces parasitoses dans le pays, en prenant en compte les informations épidémiologiques.

Translated from English version into French by Suzanne Assenat and Véronique DAUDRIX, through

TRANSLATORS
WITHOUT BORDERS

Систематический обзор и метаанализ распространённости и распределения гельминтных инфекций, передаваемых через почву, у детей в Нигерии.

Соломон Нгутор Каршима (Solomon Ngutor Karshima)

Аннотация

Справочная информация: В связи со сложностями внедрения мер по контролю, заражение гельминтными инфекциями, передаваемыми через почву (ГПП) ,по-прежнему остается существенной проблемой в странах с ограниченными ресурсами. Например, в Нигерии, несмотря на ряд имеющихся отчётов по общинам и провинциям, отсутствуют национальные данные о распространённости, тяжести, а также о зонах риска (ЗР) ГПП.

Методы: Настоящее исследование проведено в соответствии с Предпочтительными параметрами отчетности для систематических обзоров и метаанализа (PRISMA) с целью выяснения распространённости, распределения и ЗР ГПП среди нигерийских детей посредством метаанализа данных, опубликованных в период с 1980 по 2015 гг. Общая оценка распространнёности (ООР) была установлена посредством применения модели случайных эффектов, в то время как гетерогенность рассчитывалась по критерию Q Кохрена.

Результаты: В общей сложности 18 901 из 34 518 Нигерийских детей в возрасте от 0 да 17 лет, обследованных во время проведения данного исследования в 19 штатах Нигерии, были инфицированы как минимум одним видом ГПП. Общая распространённость ГПП составила 54,8% (95% *CI*: 54,2 - 55,3). Охват ГПП по подгруппам составил 13,2% (95% *CI*: 11,5; 15,1) и 80,9% (95% *CI*: 80,0; 81,7). Наиболее высокая распространённость ГПП наблюдалась среди детей в сообществах (59,0%; 95% *CI*: 57,7; 60,4), а также среди детей школьного возраста (54,9%; 95% *CI*: 54,3; 55,5). *Ascaris lumbricoides* оказался самым распространённым видом (44,6%; 95% *CI*: 44,0; 45,2). Более 36% (15/41) исследований были опубликованы в юго-западной Нигерии. Юго-западный регион был единственной зоной высокого риска (ЗВР) инфицирования ГПП, в то время как остальные регионы оказались зонами с низким уровнем риска (ЗНР).

Вывод: ГПП инфекции, связанные с заражением *Ascaris lumbricoides*, *Strongyloides stercoralis*, *Trichuris trichiura*, а также анкилостомозом широко распространены по всей Нигерии. Стратегическое применение антигельминтных препаратов, санитарное просвещение и

надлежащая санитария, с учетом данной эпидемиологической информации, помогут взять под контроль распространённость этих инфекций в Нигерии.

Translated from English version into Russian by Inna Beltcova and Liudmila Tomanek, through

Prevalencia y distribución de infecciones de helmintos transmitidos por el contacto con el suelo en niños nigerianos: una revisión sistemática y meta-análisis

Solomon Ngutor Karshima

Resumen

Antecedentes: Las infecciones por helmintiasis transmitidas por el suelo (STH, en inglés) a menudo afectan a las comunidades más pobres y necesitadas. En Nigeria, por ejemplo, a pesar de varios informes comunitarios y provinciales, aún faltan datos a nivel nacional sobre la prevalencia, las responsabilidades y las zonas de riesgo (RZ en inglés) de las infecciones de STH.

Métodos: El presente estudio utilizó las recomendaciones de los puntos preferidos de informes para revisiones sistemáticas y meta-análisis (PRISMA en inglés) para determinar la prevalencia, distribución y RZ de las infecciones de STH entre los niños nigerianos a través del meta-análisis de los datos publicados entre 1980 y 2015. La estimación de prevalencia combinada (PPE en inglés) se determinó mediante el modelo de efectos variados, mientras que la heterogeneidad se evaluó utilizando la prueba Q de Cochran.

Resultados: Un total de 18.901 niños nigerianos de edades comprendidas entre los 0 y los 17 años, de los 34.518 estudiados en los 19 estados nigerianos durante el periodo de revisión se infectaron con una o más especies de STHs. La PPE general para infecciones de STH fue de 54'8% (95 % *IC*: 54'2, 55'3). Las PPEs de subgrupos oscilaron entre el 13'2% (95% *IC*: 11'5, 15'1) y el 80'9% (95% *IC*: 80'0, 81'7). Las PPEs más elevadas para infecciones de STH se observaron en niños a nivel comunitario (59'0%, 95% *IC*: 57'7, 60'4) y en niños de edad escolar (54'9%, 95% *IC*: 54'3, 55'5). *Ascaris lumbricoides* fue la especie predominante (44'6%, 95% *IC*: 44'0, 45'2). Sobre el 36% (15/41) de los estudios publicados trataban del sudoeste de Nigeria. La región sudoeste fue la única zona de alto riesgo (HRZ en inglés) de infecciones de STH, mientras que las demás regiones fueron zonas de bajo riesgo (LRZ en inglés).

Conclusión: Las infecciones de STH relacionadas con *Ascaris lumbricoides*, *Strongyloides stercoralis*, *Trichuris trichiura* y helmintos son muy prevalentes en todo Nigeria. El uso estratégico de antihelmínticos, la educación sanitaria y una mejor higiene, teniendo en cuenta esta información epidemiológica, ayudarán a controlar dichas infecciones en Nigeria.

Translated from English version into Spanish by Carmen Blazquez and Manuela Evans, through
