# Supplementary figures and images for: Prevalence and distribution of soil-transmitted helminth infections in Nigerian children: a systematic review and meta-analysis
Source: Infect Dis Poverty. 2018 Jul 9;7:69. doi: 10.1186/s40249-018-0451-2 (PMC6036687; doi:10.1186/s40249-018-0451-2)

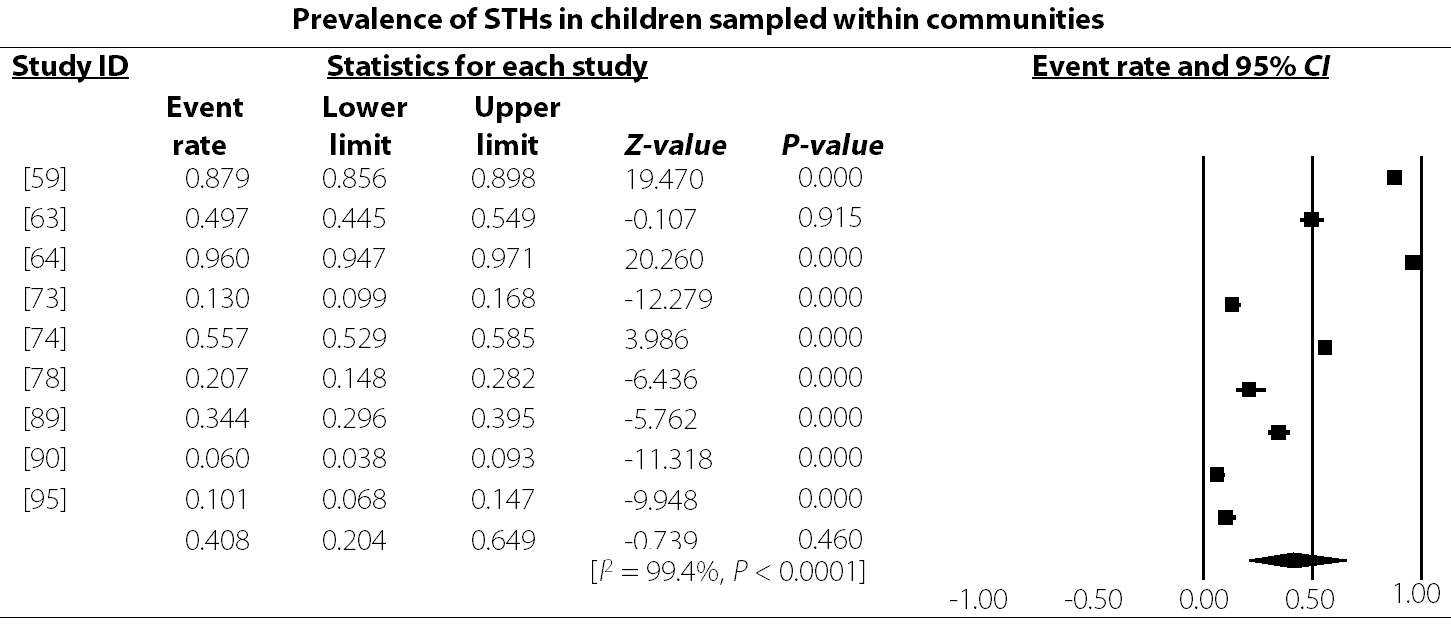

Supplement: Supplementary file 3 — Forest plot for the prevalence of STHs in children sampled within communities. (DOCX 77 kb) [file 40249_2018_451_MOESM3_ESM.docx]

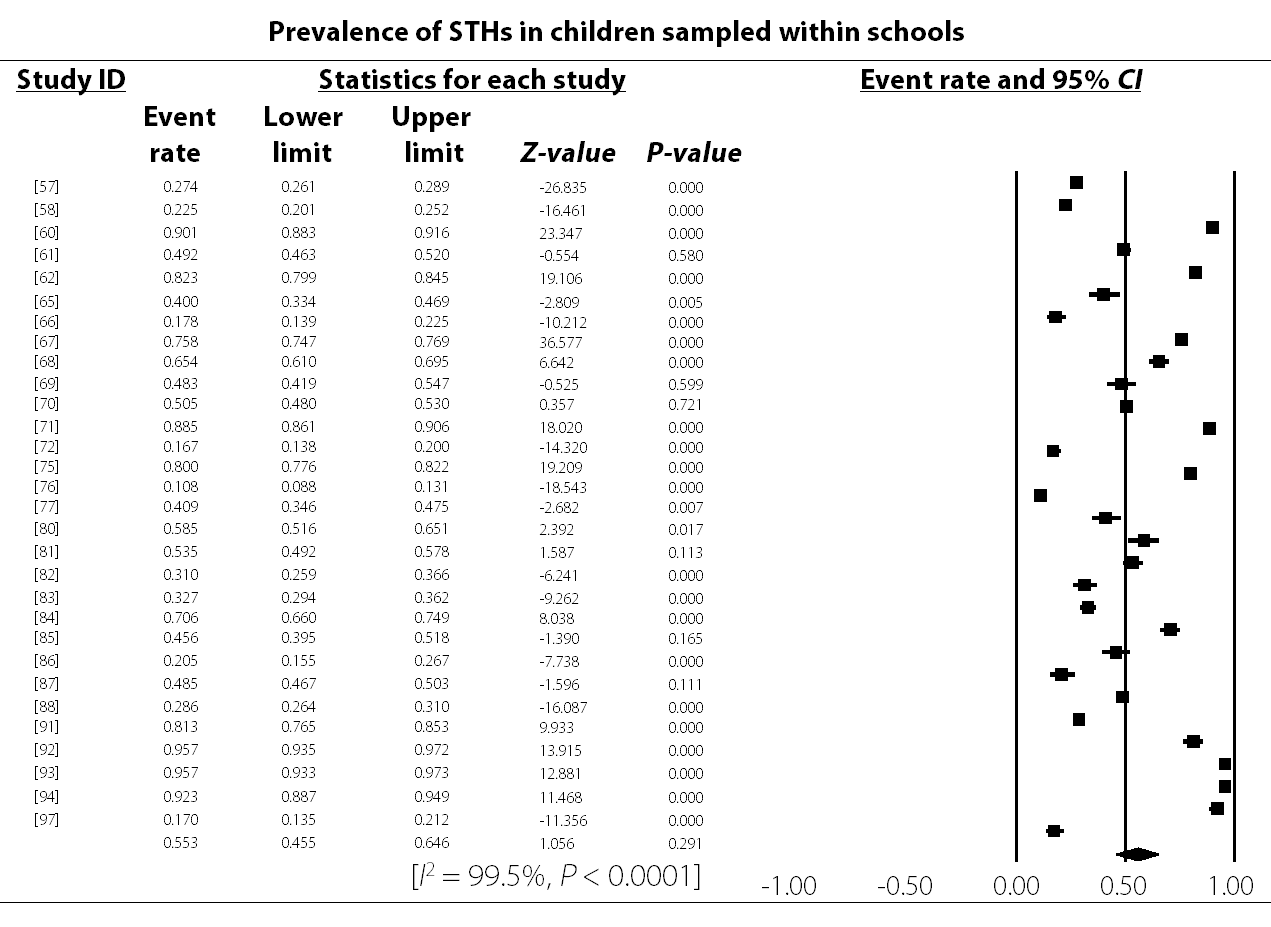

Supplement: Supplementary file 4 — Forest plot for the prevalence of STHs in children sampled within schools. (DOCX 95 kb) [file 40249_2018_451_MOESM4_ESM.docx]

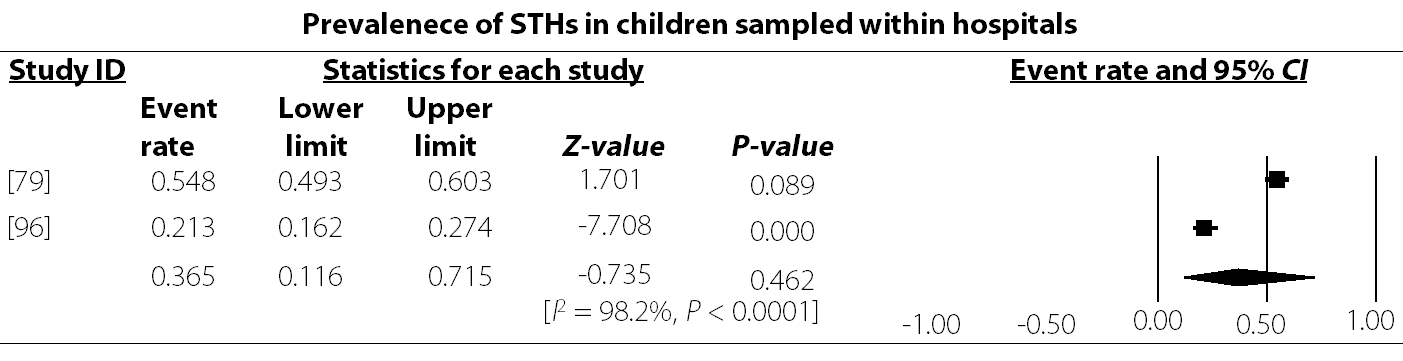

Supplement: Supplementary file 5 — Forest plot for the prevalence of STHs in children sampled within hospitals. (DOCX 50 kb) [file 40249_2018_451_MOESM5_ESM.docx]
